# Evidence-based practice strategies for faculty professional development in clinical translational research at Institutional Development Award (IDeA) state institutions in the Mountain West

**DOI:** 10.1017/cts.2022.407

**Published:** 2022-05-20

**Authors:** Merle R. Kataoka-Yahiro, Larissa Myaskovsky, Ruben K. Dagda, Akshay Sood, Kathrene Conway, Joseph Guerrero Lopez, Mark R. Burge, Francisco S. Sy

**Affiliations:** 1 Nancy Atmosphera-Walch School of Nursing, Department of Nursing, University of Hawaii at Manoa, Honolulu, HI, USA; 2 Center for Healthcare Equity in Kidney Disease and Department of Internal Medicine, University of New Mexico Health Sciences Center, Albuquerque, NM, USA; 3 University of Nevada, Reno, School of Medicine, Reno, NV, USA; 4 Miners’ Colfax Medical Center Endowed Chair in Mining-Related Lung Diseases, Miners’ Wellness Tele-ECHO Clinic, Mentoring and Faculty Retention, UNM School of Medicine, Office of Faculty Affairs and Career Development, Division of Pulmonary, Critical Care and Sleep Medicine, Department of Internal Medicine, University of New Mexico School of Medicine, Albuquerque, NM, USA; 5 School of Public and Community Health Sciences, University of Montana, Missoula, MT, USA; 6 MW CTR-IN Program, School of Public Health, University of Nevada, Las Vegas, NV, USA; 7 Department of Medicine, Endocrinology & Metabolism, University of New Mexico, Albuquerque, NM, USA; 8 Department of Environmental and Occupational Health, Mountain West Clinical & Translational Research Infrastructure Network (CTR-IN), University of Nevada Las Vegas (UNLV), School of Public Health, Las Vegas, NV, USA

**Keywords:** MW CTR-IN, clinical translational research, mentoring, career development, research education & training, health disparities

## Abstract

The Mountain West Clinical Translational Research – Infrastructure Network (MW CTR-IN), established in 2013, is a research network of 13 university partners located among seven Institutional Development Award (IDeA) states targeting health disparities. This is an enormous undertaking because of the size of the infrastructure network (encompassing a third of the US landmass and spanning four time zones in predominantly rural and underserved areas, with populations that have major health disparities issues). In this paper, we apply the barriers, strategies, and metrics to an adapted educational conceptual model by Fink (2013). Applying this model, we used four tailored approaches across this regional infrastructure network to: (1) assess individual faculty specific needs, (2) reach out and engage with faculty, (3) provide customized services to meet the situational needs of faculty, and (4) utilize a “closed communication feedback loop” between Professional Development (PD) core and MW CTR-IN faculty within the context of their home institutional environment. Summary statement results from participating faculty show that these approaches were positive. Grounded in best educational practice approaches, we have an opportunity to refine and build from this sound foundation with implications for future use in other CTR-IN networks and institutions in the IDeA states.

## Introduction

**Fig. 1.**
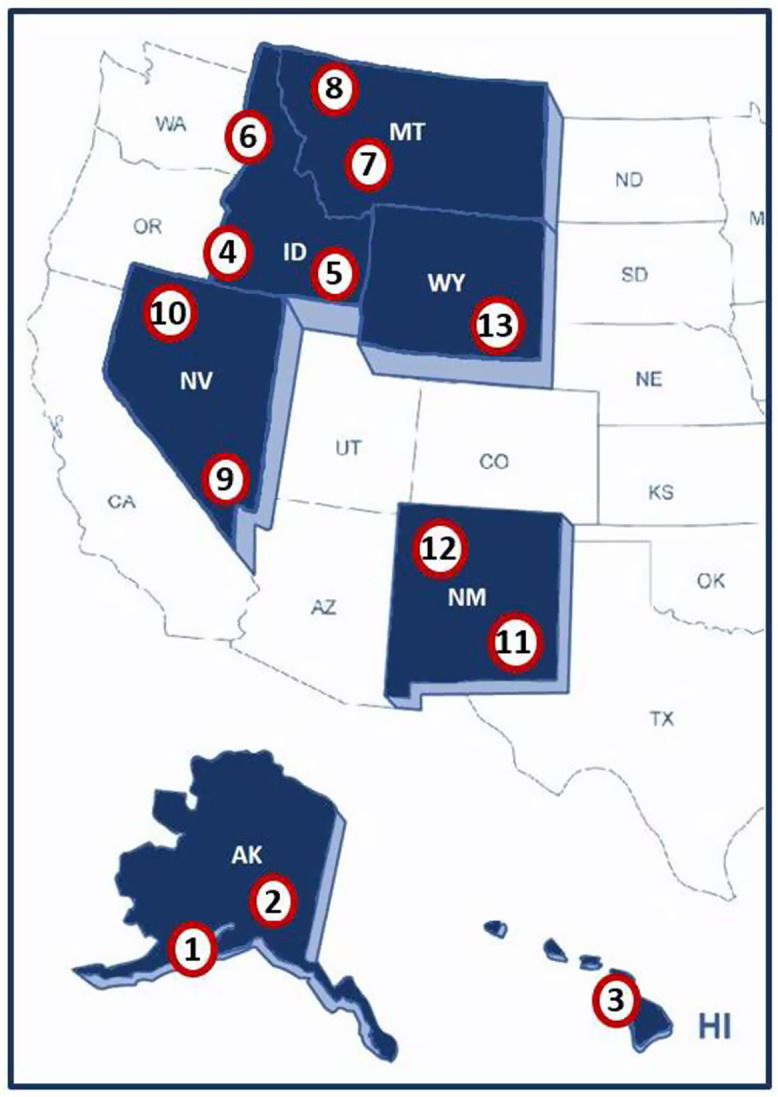
Mountain West CTR-IN program university partners. **Legend: AK –** Alaska: (1) University of Alaska, Anchorage, (2) University of Alaska, Fairbanks; **HI – Hawaii:** (3) University of Hawaii at Manoa (*Professional Development (PD) Core*); **ID – Idaho:** (4) Boise State University (Boise), (5) Idaho State University (Pocatello), (6) University of Idaho (Moscow); **MT – Montana:** Montana State University (Bozeman), (8) University of Montana (Missoula) (*Clinical Pilots Project Program (CP3) and Community Engagement & Outreach (CEO) Core(s)*); **NV – Nevada:** (9) University of Nevada, Las Vegas - ***Host Institution*** (*Administrative Core*), (10) University of Nevada, Reno – School of Medicine - (*PD and Biostatistics, Epidemiology, Research & Design (BERD) Core(s)*); **NM – New Mexico:** (11) New Mexico State University (Las Cruces), (12) University of New Mexico – School of Medicine (Albuqurque) - (*PD, BERD, and Tracking & Evaluation (T&E) Core(s)*); **WY – Wyoming:**University of Wyoming (*CP3 Core*) (13).

The Mountain West Clinical Translational Research – Infrastructure Network (MW CTR-IN) is hosted at the University of Nevada, Las Vegas and supported by the National Institutes of Health (NIH) through the National Institute of General Medical Sciences (NIGMS) Award #1U54GM104944-01A1 under the Institutional Development Award (IDeA) program. Established in 2013, MW CTR-IN is a research network of 13 university partners located among seven IDeA states targeting health disparities (see Fig. 1). These states are designated as IDeA states because they have historically received low levels of funding from the NIH. The university partners include the University of Alaska – Anchorage, University of Alaska – Fairbanks (1,2), University of Hawaii – Manoa (University of Hawaii at Manoa, John A Burns School of Medicine (3)), Boise State University, Idaho State University, University of Idaho (4-6), Montana State University, University of Montana (7-8), University of Nevada Las Vegas (host) (University of Nevada – Las Vegas, Kirk Kerkorian School of Medicine at UNLV), University of Nevada Reno (University of Nevada – Reno – School of Medicine (9-10)), New Mexico State University, University of New Mexico (University of New Mexico School of Medicine (11-12)), and the University of Wyoming (13). A unique feature of the MW-CTR-IN Network is that it encompasses a third of the US landmass-spanning four time zones in predominantly rural and underserved areas, with populations that have major health disparities issues [1–3].

**Fig. 2.**
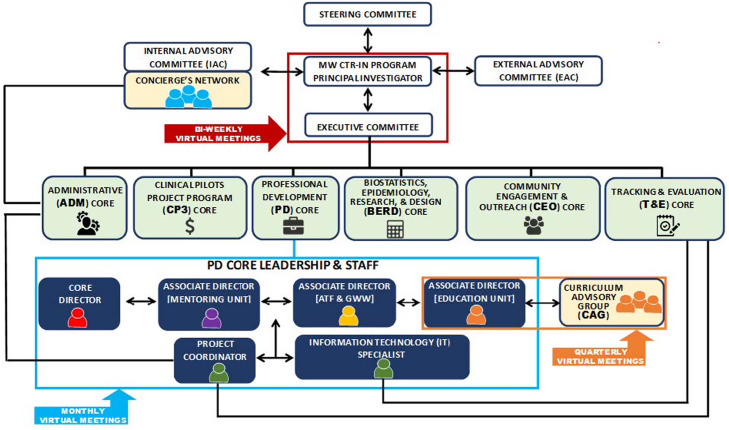
Organizational structure – MW CTR-IN program & PD core communications. **Legend: ADM –** Administrative Core; **ATF –** Advance to Funding Programs; **BERD –** Biostatistics, Epidemiology, Research, & Design Core; **CEO** – Community Engagement & Outreach Core; **CP3 –** Clinical Pilots Project Program Core; **CAG –** Curriculum Advisory Group; **EAC –** External Advisory Committee; **GWW –** Grant Writing Workshop; **IT –** Information Technology; **IAC –** Internal Advisory Committee; **PD** – Professional Development Core; **T&E** – Tracking & Evaluation Core.

Yet, despite these dauting challenges, we have successfully created and implemented a highly functional virtual and effective CTR-IN research network in the past 8 years due to the efforts of the MW CTR-IN research network consisting of an experienced and effective team of Core Directors and Associate Directors located at 6 University hubs (University of Nevada, Las Vegas, University of Nevada, Reno, University of Hawaii at Manoa, University of Montana, and University of New Mexico, Albuquerque, University of Wyoming), encompassing nearly half of the 13 MW universities. Our MW CTR-IN organizational structure provides a strong, effective, and collaborative governance of our 13 MW University Partners (i.e., Internal Advisory Committee (IAC), External Advisory Committee (EAC), Executive Committee (EC), Steering Committee (SC)). The MW CTR-IN program is also a time tested highly functional and effective organizational structure consisting of our 4 original required cores and two new required cores: Community Engagement & Outreach (CEO) and Tracking and Evaluation (T& E) (see Fig. 2).

**Fig. 3.**
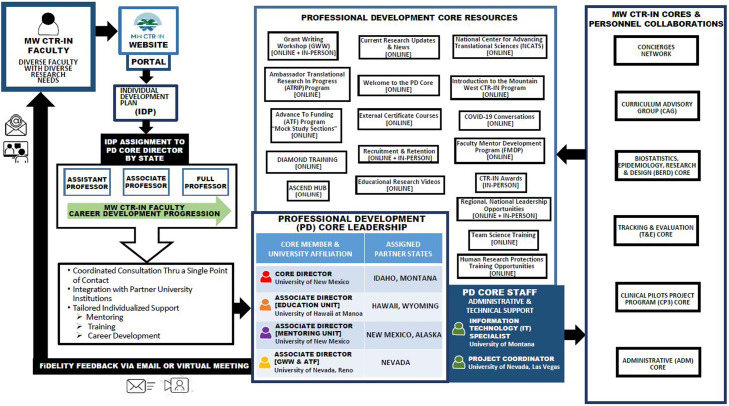
Professional Development Core – Communication Linkage through the MW CTR-IN Portal & Portfolio. **Legend: ADM –** Administrative Core; **ATF –** Advance to Funding; **ATRIP –** Ambassador Translational Research in Progress; **BERD –** Biostatistics, Epidemiology, Research, & Design Core; **CAG –** Curriculum Advisory Group; **CP3 –** Clinical Pilot Projects Program Core; **CTR-IN –** Clinical Translational Research Infrastructure Network; **FiDeLITY** – Frequent, Immediate, Discriminating, Loving; **FMDP –** Faculty Mentor Development Program; **GWW –** Grant Writing Workshop; **IDP** – Individual Development Plan; **IT –** Information Technology; **MW CTR-IN** – Mountain West Clinical Translational Research Infrastructure Network; **NCATS –** National Center for Advancing Translational Sciences; **PD –** Professional Development Core; **T&E –** Tracking and Evaluation Core.

The Professional Development (PD) core is led by a Director and three Associate Directors, Information Technology (IT) Specialist, and Administrative Specialist. The group meets monthly to discuss agenda issues and updates on PD core work. The Core also meets with the MW CTR-IN EC on a bi-weekly basis. It is very common for the PD core to receive and send multiple emails to and/or meet remotely via Zoom or similar conference web-based platforms with each other on PD core issues. The activities of the PD core are highly significant for the overall goals of the MW CTR-IN program as they provide innovative strategies to reach the faculty of the 13 institutions in efforts to increase the number of research teams with capacity and competency building to address regional health disparities. During the new funding renewal period (years 6–10), the PD core refined its CTR assessment and mentoring program for ease of access, with rapid response times for investigators distributed across our network. In preparation for the renewal, the PD core Director and Associate Directors were involved with reviewing the reports of needs assessments, focus group interviews, and meeting minutes completed in years 1–5. Sessions were held and feedback obtained from the pilot awardees, concierges (university representatives assigned to assist pilot awardees), EC, IAC, EAC, PI annual visits to MW CTR-IN institutions, and annual meeting minutes between PD core and pilot awardees and MW CTR-IN faculty and staff and EC cores. The summation of all reports and findings led to the implementation of the PD core’s new philosophy of “providing whatever specific investigators need, whenever they need it, and in a way that is convenient for them.” (see Fig. 3).

The purpose of this conceptual paper is to describe the framework, evidence-based approach, and educational methods and strategies utilized to provide regional faculty development within the MW CTR-IN. In applying an educational conceptual framework, PD core members used four tailored approaches across this regional infrastructure network to: (1) assess individual faculty specific needs, (2) proactively reach out and engage with faculty, (3) provide customized services to meet the situational needs of faculty, and (4) utilize a “closed communication feedback loop” between PD core and MW CTR-IN faculty within the context of their home institutional environment.

Mentoring, training, resources, and services are central to producing research that will contribute to sustained improvement in health within our communities and for retaining promising early investigators in the biomedical workforce. Our network includes faculty who have different demands on their time for other academic responsibilities. Institutional resources specific to CTR training and career development are heterogeneous across our network. An understanding of the barriers of faculty, strategies to meet the faculty needs, and capturing metrics by the PD core, are critical in helping MW faculty overcome these challenges to develop and maintain successful research programs (see Table 1).

**Table 1.** Barriers, conceptual model, evidence-based practice strategies, & metrics

*1. Provide expert consultation and mentoring to CTR investigators across our network*			
Barriers	Conceptual Model: Applying Concepts of Significant Learning – Fink (2013)	Evidence-based Practice Strategies	Metrics
– Need for timely, comprehensive, expert consultations	FiDelity Feedback	– Single point of contact for rapid consultation and identification of appropriate resources	Number of PD consultations for investigators; turnaround times Number of IDPs created for investigators engaged in different types of CTR Number of formal mentoring relationships established and maintained Number of just-in-time mentoring activities Improved research productivity, as measured by publications and grants
– Different professional development needs for different members of CTR Teams	In-depth Situational Analysis; Situational Factors	– IDPs tailored to individual faculty development needs – ATRIP monthly topics selected based on institutional interest at MW CTR-IN institutions	
– Need for flexible, effective CTR mentoring	Active Learning; Educative Assessment	– IDPs tailored to individual faculty mentoring needs – Effective FMDP – Long-term or just-in-time mentoring (based on individual needs of unsuccessful pilot award applicants, current pilot awardees, former pilot award recipients, pilot awardees with extramural funding, for all other research faculty)	

*2. Tailoring and providing access to educational research resources required for CTR faculty.*			
Barriers	Conceptual Model: Applying Concepts of Significant Learning – Fink (2013)	Evidence-based Practice Strategies	Metrics
– Need to integrate activities with locally available resources	Teaching and Learning Activities	– Partnership with COBRE & INBRE core facilities – ATRIP integration with local faculty to present topics relevant to their State/Region – Updated list of complementary resources at partner institutions and coordination with concierges – Recruitment of local faculty for CAG – Recruitment of local faculty facilitators and attendees in the FMDP	Number of courses and training sessions available through the PD Core Numbers of unique users of content, via PD portal and face-to-face Number of GCP-trained individuals on IRB protocols Number of CTR-IN investigators trained in COBRE and INBRE facilities Number of new course work and training modules specific to regional disparities research Number of participants among CTR staff, students, and community stakeholders Number of collaborative activities with other IDeA programs, as measured by use of cores and educational resources by CTR-IN investigators
– Lack of critical mass of researchers at individual institutions	Situational Factors; Educative Assessment	– Training new and existing researchers using resources provided by CAG ATRIP FMDP	
– Different educational content requirements for different members of CTR teams – Need for course work and training in disparities research – Lack of institutional funding for course fees – Lack of connection to other IDeA resources in network	Situational Factors; Educative Assessment: Teaching and Learning Activities-Feedback & Assessment-Learning Goals	Promoting access and utilization of MW-CTR-IN educational resources by CAG: – Promoting synchronous and asynchronous distance learning – Extensive leveraging of existing content through: Negotiation of shared use agreements and support of licensing to provide free access to course work and training Development of course work and training modules specific to regional opportunities and needs Support of course work and training opportunities in INBRE and COBRE facilities	

*3. Provide training and support services required for sustaining a career in CTR at MW institutions.*			
Barriers	Conceptual Model: Applying Concepts of Significant Learning – Fink (2013)	Evidence-based Practice Strategies	Metrics
– Heterogeneity in protected time for research and training	In-Depth Situational Analysis; Situational Factors	– Training mentors on mentees’ time management (FMDP) – Emphasis on asynchronous distance-based learning (CAG) – Identifying specific CTR content needed (such as budget management) (ATRIP, GWW) – Networking between like-minded faculty (ATRIP)	Number of participants in GWWs and mock study sections Number of successful grant applications and competitive renewals facilitated by the PD Core Number of CTR teams identified on CTR-IN and institutional websites Number of CTR-IN research awards distributed to team members across network Adoption of harmonized tenure and promotion guidelines recognizing CTR and Team Science at all network institutions Numbers represented on regional and national leadership committees
– Limited institutional recognition of impact and value of CTR contributions	In-Depth Situational Analysis; Situational Factors	– Recognition of contributions by leaders, mentors, and pilot awardees at the Annual Meeting and on the website	
– Limited role models for successful careers in CTR	In-Depth Situational Analysis; Situational Factors	– Institutional training in Team Science – Mock study sections for training as a peer reviewer & grant applicant (GWW) – Training mentors (FMDP) – Networking with role models (Annual Meeting, ATRIP)	
– Limited understanding of national review and dissemination approaches for CTR	In-Depth Situational Analysis; Situational Factors	– Frequent regional MW CTR-IN GWW	
– Limited opportunities for regional and national leadership in CTR	In-Depth Situational Analysis; Situational Factors	– Leadership opportunities at COBRE, INBRE, CTSA; Executive Steering Committee of the MW CTR-IN; External CTR-IN Advisory Committees-Leadership at NRMN (FMDP)	

Legend: ATRIP – Ambassador Translational Research in Progress; FMDP – Faculty Mentor Development Program; CAG – Curriculum Advisory Group; COBRE – Centers of Biomedical Research Excellence; CTR – Clinical Translational Research; CTR-IN – Clinical Translational Infrastructure Network; Fidelity – Frequent, Immediate; GCP – Good Clinical Practice; GWW – Grant Writing Workshop; IDeA – Institutional Development Award; IDP – Individual Development Plan; INBRE – IDeA Networks of Biomedical Research Excellence; MW CTR-IN – Mountain West Clinical Translational Research Infrastructure Network; NRMN – National Research Mentoring Network; PD – Professional Development Core.

## Conceptual Framework

**Fig. 4.**
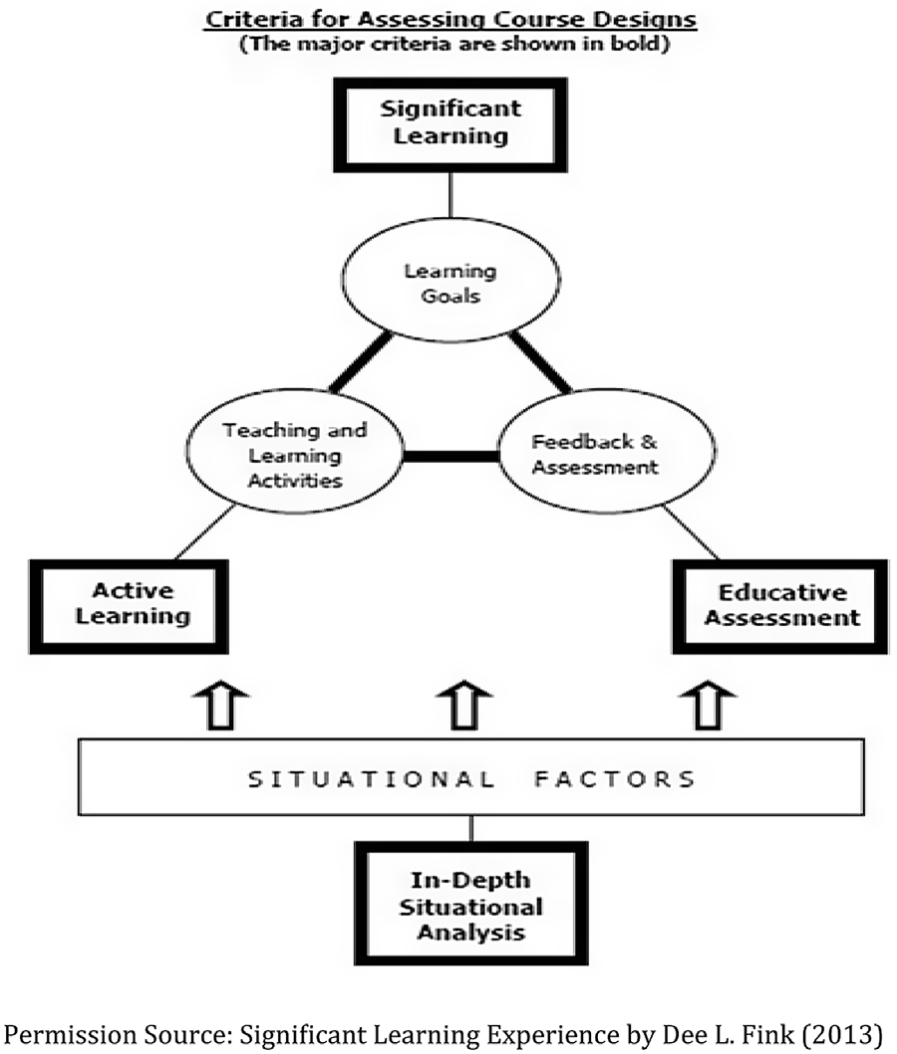
Significant learning experience – criteria.

Our educational ***guiding principle*** is to individualize support to each faculty researcher, within specific institutional environments, with different competing time demands, and career goals. This guiding principle is foundationally grounded in epistemology, based on Significant Learning Experiences [4]. The goals of the PD core align with the concepts described in Fink’s work to integrate significant learning experiences (see Fig. 4). The use of active learning and “educative assessment” are utilized. Educative assessment includes forward-looking assessment (looking ahead of what learners are expected to do or what learners are able to do in the future as the result of having learning about x, y, and z), self-assessment, criteria and standards, and FIDeLity feedback. FIDeLity is part of Educative Assessment and this feedback needs to be frequent, immediate, discriminating [based on clear criteria and standards] and with supportive constructive delivery which is a fundamental component to the PD core’s new philosophy.

FiDelity feedback includes frequent and immediate feedback along with providing constructive criticism in an empathetic way to the MW CTR-IN faculty. It is essential to understand the situational factors in creating significant learning experiences for faculty. Therefore, the input and collaboration at the home institution is critical to the success of each faculty. The PD core emphasizes continual lifelong learning by helping faculty investigators engage in their learning.

The PD core combines both traditional and modern paradigms which embrace new methods of teaching and learning. The old paradigm [5] includes knowledge which is transferred in a linear way. Learners are passive “vessels” to be filled by mentor’s knowledge. The traditional mode of learning is memorizing, where growth and goals are only to complete requirements and to achieve certification within a discipline. The relationships are mostly impersonal and tacit among mentors and mentees, and the context and climate are competitive, individualistic, and conforming. In the new paradigm, the PD core is highly proactive by being attentive to the feedback of the learner (the MW CTR-IN faculty), with greater attention to the experience rather than the information delivered. Although foundational knowledge is important, each faculty’s experience and growth are equally, or more, important to the success and desired outcome of each investigator.

## Evidence-based Practice Strategies

The three tailored approaches adapted from the educational conceptual model by Fink (2013) are linked to areas of evidence-based practice strategies to address barriers and evaluation of metrics. Strategies successfully incorporated into our MW CTR-IN PD core are (1) providing expert consultation and mentoring to CTR investigators across the network, (2) tailoring and providing access to educational CTR research resources required by CTR faculty, and (3) providing training and support services required for sustaining a career in CTR at MW institutions.

### Providing Expert Consultation and Mentoring to CTR Investigators Across the Network

An important faculty barrier includes the need for timely, comprehensive, personalized expert consultation on varying professional developmental needs based on diverse capacities and areas of expertise. Approaches to address this barrier include having a single point of contact for rapid consultation and identification of appropriate resources, individual development plans (IDPs) tailored to individual faculty with different levels of experience and research tracks, and long-term or just-in-time (real-time) mentoring.

*Each PD Core director (includes Director and three Associate Directors) adopts a partner institution(s)*. PD core directors are assigned to two or three MW CTR-IN participating states, within which each director has the responsibility to communicate with partner investigators, research concierges (institutional point of contact administrative staff), and other CTR-IN cores. This process improves communication, assessment, and feedback, establishes criteria and standards of mentoring and training, and enhances rapport, trust, and continuity of partner institution faculty with the PD core. In addition, all communication with MW faculty is logged within the member only online database portal, so information is easily accessible between all the various cores.

*PD core web portal becomes an entry point for all MW CTR-IN tenured and tenure-leading faculty* so that faculty become familiar with the MW CTR-IN PD core criteria, standards, and resources. Through instant email contacts via the MW CTR-IN internet site link (https://ctrin.unlv.edu/), faculty receive near-immediate feedback by PD core leadership and staff. The support is easily accessible, and individually customized, including resources for mentoring, education, and career development.

The PD core uses a decentralized approach’ which is based on individual institution partner assets and investigator situational factors and needs. The PD core director assigned to a specific faculty member collaborates with the research concierges at their assigned partner institutions to identify and maintain up-to-date listings on the PD core website of consultation, mentoring, and training resources available at each of the 13 institutions. This type of information helps reduce duplication of effort.

Through this approach of “personal diplomacy” PD core leadership has made meaningful connections with their respective constituents to encourage participation in PD core activities. The MW CTR-IN portal has been overhauled to act as a seamless informational system that can track and monitor most activities for each core. Interactions between applicants and MW CTR-IN are captured and monitored for customer satisfaction. The various tasks in the portal, including monitoring and evaluation of individual interactions, are open to MW CTR-IN faculty. The various MW CTR-IN cores can access materials (didactic logs) to ensure that duplicate work between cores is minimized, thereby enhancing collaborations in the portal. Customer satisfaction with every interaction that occurs is being tracked with every PD core member (and other cores). In 2020–2021, MW CTR-IN faculty satisfaction data with interaction with PD core members collected show that the PD core has been rated with 100% in the ‘strongly agree’ category with interactions with MW CTR-IN faculty based on nine interactions.

### Tailoring and Providing Access to CTR Educational Research Resources Required by Faculty

Barriers that CTR faculty face include the need to integrate activities with locally available resources within their institutions, lack of critical mass of funded researchers at individual institutions, different educational content requirements for different members of CTR teams, need for course work and training in health disparities research, a lack of institutional funding for course fees, and lack of connection to other IDeA resources in the network. The CTR education research resources (a) tailors to the educational/career development needs for each region/state, (b) includes an orientation video that introduces the services of the PD core to CTR faculty, (c) supports mentoring and networking cohort building with synchronous and asynchronous distance learning, (d) includes extensive leveraging of existing CTR content within and outside of the network, (e) supports course work and training opportunities in IDeA Networks of Biomedical Research Excellence (INBRE) and Centers of Biomedical Research Excellence (COBRE) research core facilities, and (f) shares use agreements and support of licensing to provide free access to course work and training to CTR-IN investigators by University of Nevada, Las Vegas, University of New Mexico, and University of Hawaii at Manoa.

The PD core has established a PD Curriculum Advisory Group (CAG) to provide valuable feedback on curriculum design and educational content. PD core members work closely with the CAG members who are representative of the network. This group is composed of eight to ten network representatives appointed by the PD core Director and have significant content expertise in health disparities – related research and or curriculum development. Members serve a two-year voluntary appointment and meet quarterly via Zoom to formally recommend courses/training/topics/teaching and learning strategies that address (a) competencies appropriate for CTR, (b) content and presentation of excellent quality, and (c) access and availability to MW CTR-IN faculty.

In addition to the input from the CAG, all PD core education research resource trainings are tracked for usage. These metrics help provide information about which courses are being used and which ones are not. In addition, evaluation questions are asked after faculty begin the courses to provide additional feedback about the quality and usefulness of the materials. Relevant PD upcoming events are published through a “PD Core Newsflash,” a one-page PDF document that is distributed widely through the concierges of all MW CTR-IN institutions and published on a bi-monthly basis on new PD core resources and highlights.

Based on feedback from the PD core, CAG, and other cores, a broad range of education research resources includes external certificates, short courses, and current research updates on topics pertaining to (1) Health Disparities Research, (2) Regulatory Issues & Ethics, (3) Clinical Research, (4) Team Science, (5) Informatics, (6) Grantsmanship, and (7) Career Development. We deliver multifaceted, yet specific content driven topics for our MW CTR-IN faculty. The educational continuum stretches from traditional credit-based graduate program certificate and degree completion courses, web-based non-degree certificate-based courses, short courses, and webinars, and news and updates. In other words, there is something for every MW CTR-IN faculty. The top 10 education research resource course views this year include – orientation PD core educational resource orientation training, DIAMOND training (collection of rich materials for researchers in IDeA, CTR-IN, COBRE, & CTSA programs), Human Research Protection, COVID19 Conversations, NIH Accelerating Solutions for Commercialization & Entrepreneurial Development (ASCEND HUB), National Center for Advancing Translational Sciences (NCATS) MEDI 501: Principles of Preclinical Translational Science, and Translational Science 2021 – Proposals and Abstracts. In Table 2, displays the comparison of education research resource courses with or without registered tickets opened by MW CTR-IN faculty, courses with submitted tickets by university, and courses with submitted tickets by unique individual faculty by university. There were 219 education research resource courses opened in MW CTR-IN PD core portal, tickets submitted to the PD core by university were a total of 162 and 125 unique individual (faculty) submitted tickets in 2020–2021 (see Table 2).

**Table 2.** Impact of the PD core by university including courses opened and tickets submitted

MW CTR-IN Universities	Courses Opened in MW CTR-IN Website	Tickets Submitted to the PD Core By University	Tickets Submitted by Unique Individuals (Faculty)
Boise State University	6	9	9
Idaho State University	16	18	11
Montana State University	2	5	4
New Mexico State University	3	18	10
University of Alaska, Anchorage	2	14	12
University of Alaska, Fairbanks	2	5	4
University of Hawaii at Mānoa	93	11	11
University of Idaho	6	9	7
University of Montana	17	4	4
University of Nevada, Las Vegas	13	23	18
University of Nevada, Reno	19	28	19
University of New Mexico	33	6	5
University of Wyoming	3	12	11
GRAND TOTAL	219	162	125

**Legend: CTR-IN** – Clinical & Translational Research Infrastructure Network; **MW CTR-IN** – Mountain West Clinical & Translational Research Infrastructure Network; **PD** – Professional Development Core.

### Providing Training and Support Services Required for Sustaining a Career in CTR at MW Institutions

Another challenge for faculty in the MW region is that each MW CTR-IN member institution has variable (1) protected time for research and training among faculty, (2) institutional recognition of impact and value of CTR contributions, (3) number of mentors and role models for successful careers in CTR, (4) an understanding of review of extramural research grants and dissemination approaches for CTR, (5) opportunities in regional and national leadership in CTR, (6) support for recruitment and retention of key CTR team members, and (7) rates of faculty attrition. Strategies to address this variability include providing (1) regional CTR Grant Writing Workshop (GWW)s, (2) rigorous mock study sections – the Advance to Funding (ATF) Program, (3) the Ambassador Translational Research in Progress (ATRIP) Program, and (4) the Faculty Mentor Development Program (FMDP) and National Research Mentoring Network (NRMN) resources. Such programs help MW CTR-IN institutions enhance the career development of a diverse scientific and educator workforce for MW CTR-IN faculty at their institutions.

The GWW and the ATF Programs are two innovative career development services offered by the PD core of the MW CTR-IN program. In brief, the GWW is targeted toward junior to mid-career CTR investigators from any of the 13 participating MW CTR-IN institutions who need specific and productive feedback on developing extramural research grant applications [i.e., National Institute of Health (NIH), National Science Foundation (NSF), or Department of Defense (DOD)]. Participants receive mentoring and continuous feedback by attending one of two GWWs tailored to CTR focused on health disparities offered each year. One workshop is dedicated to new investigators with no significant extramural funding. A second workshop is tailored to advanced grant seeking applicants who have preliminary data and have received significant intramural funding. These GWWs are not static because applicants and their mentors receive extensive feedback during interactive sessions to help them improve a draft of their specific aims page and research strategy. In 2018–2019, more than 27 attendees from 8 institutions have participated in the GWW, with an overall satisfaction of 4.5 (range: 1–5, 5 = Excellent). In 2021, due to the COVID19 pandemic, the GWW was successfully delivered to six participating faculty from five institutions of the Mountain West with an overall satisfaction of 4.8 (range: 1–5, 5 = Excellent). It is worth noting that 100% of the faculty that participated in the GWWs (2018–2021) completed the pre and post-evaluation surveys (*n* = 33). This is due to the fact that participation in the post-evaluation survey was mandatory so that participating faculty can receive the course material of the GWW electronically.

The ATF program is an external grant review service offered predominantly online through the MW CTR-IN portal that provides both static and real-time mentoring and critique to the applicants with at least five weeks of anticipation prior to a grant submission deadline. In addition, to overcome the frustrating process of addressing written reviews the ATF provides an NIH “style” mock study section review of the research application. This is a one-hour session to provide face-to-face feedback to go over the weaknesses and strengths of the scientific and formatting aspects of the application and provide best practices to approach a revision. Each applicant is allowed up to two rounds of reviews for each application, if time permits, to revise their proposal per reviewers’ concerns prior to the grant submission deadline. To date, the ATF has established a record of more than six funded extramural awards (totaling $16 M in funding predominantly derived from four Mountain West academic institutions including one $10 million COBRE award from 31 grants reviewed over the entire life of the CTR-IN network). The awarded institutions were Boise State University, University of Alaska, Anchorage, University of Alaska, Fairbanks, and University of Nevada, Reno.

The ATRIP Program is aimed at providing a supportive environment for developing CTR competencies and cultivates and supports MW CTR-IN faculty at individual MW CTR-IN institutions. This program was designed to (1) expand a peer group of clinical and translational scientists at MW CTR-IN who serve as role models in increasing and accelerating CTR activities in their respective MW CTR-IN institutions; (2) serve as a forum for former pilot grant awardees and other research investigators to collaborate with other “on-campus” and inter-institutional researchers on common research projects; (3) serve as a forum for early stage investigators to discuss their research in progress, research proposals, enhance their skills in CTR, and obtain feedback from their peers and senior faculty with expertise in clinical and translational science; (4) help early stage investigators learn the finer points of scientific communication and clinical research including ethics, CTR content, presentation skills, leadership attributes, legal and safety knowledge as well as common statistical and basic science technology; and, (5) establish and enhance a peer-group of clinical and translational scientists in the MW CTR-IN network.

Participants meet monthly to discuss their research, research ethics, and other topics related to conducting clinical and translational research. ATRIP is a monthly one-hour video conference session consisting of a 30-minute research-in-progress presentation and a 30-minute didactic session. Ambassadors who are former pilot awardees from MW CTR-IN are in attendance; however, other faculty interested in participating in the monthly session are welcome to join. To ensure support from participants’ Department Chairs or College Dean, a written list of expectations details the commitments of all parties to the MW CTR-IN ATRIP Program. The participant commits to participate in the ATRIP program for at least one year and to attend a minimum of eight of the 10 sessions each academic year. In addition, the participant is required to make a presentation and to actively participate in discussions. ATRIP is coordinated by the PD core leadership and administered by the MW CTR-IN administrative team. Examples of monthly topics relevant to CTR include NIH Clinical Trial Definitions, Best Practices in Mentoring Faculty, and Power Analysis and Sample Size Determination as well as “research in progress” presentations by the MW CTR-IN faculty to further increase and enhance collaborative research opportunities. Over the two years of the program, more than 61 unique attendees from 9 institutions have participated in the online ATRIP Program with an overall satisfaction of 4.37 (range: 1–5, 5 = Excellent). States which participated in the ATRIP Program were University of Idaho, Idaho State University, Boise State University, New Mexico State University, University of New Mexico, Albuquerque, University of Nevada, Las Vegas, University of Nevada, Reno, University of Montana, and University of Hawaii at Manoa.

The effectiveness of training mentors (without preselection for their research skills) to support faculty mentees in scholarly activities was recently established at the University of New Mexico Health Sciences Center and extended to MW CTR-IN institutions in 2020 [6]. Unfortunately, skilled mentors are in short supply, particularly at MW CTR-IN institutions [7]. A recent study indicated that mentor training programs and materials are the most important component of an organizational mentoring climate [8]. The Faculty Mentor Development Program (FMDP), as part of an NIH-funded National Research Mentoring Network (NRMN)-sponsored study, has multiple components to help develop effective mentors for scholarship for faculty mentees. The online asynchronous coursework component of the FMDP is supplemented by a synchronous workshop component, which was changed to video format during the pandemic. Without direct solicitation of these individual groups, the FMDP disproportionately attracts women and racial/ethnic faculty mentors and has shown to have improved self-assessed knowledge and mentoring skill, respectively [9]. A recently launched tele-mentoring program (i.e., Mentoring Networks teleECHO) provides additional training to faculty mentors on building developmental networks and enhancing skills in networking, and high quality connections. In 2020–2021, the number of faculty participants for Part 1 – Online Asynchronous Coursework was *n* = 28, Part 2 – Video Synchronous Workshops was *n* = 13, and Part 3 – TeleECHO Mentoring Networks was *n* = 6.

### Lessons Learned and Recommendations

Faculty at MW CTR-IN institutions need multidimensional faculty education and career development programs but have limited local resources. Pooling of resources through the MW CTR-IN allows for cost efficiency, use of best practice and innovative educational teaching strategies, inter-institutional networking, and use of technology for cross-collaboration and exchange of ideas. The digital transformation of everyday faculty life has made it easier to use virtual PD Core resources but barriers remain. These include lack of familiarity with resources, differences in institutional climate and culture with varying levels of “buy-in,” wide variability of faculty needs, skill sets, and utilization patterns, and limited data on medium-term and long-term effectiveness of interventions. Lessons learned include need for representative leadership, combining virtual outreach with physical outreach strategies, advertisement of resources using a combination of newsletters, websites and social media sites, technology education and orientation, building long-term partnerships within and across institutions, and use of standard platforms for data collection.

Given these challenges, the MW CTR-IN PDC has accomplished to address many of the barriers in a short time, despite the COVID19 pandemic. Most of our outreach efforts continued successfully online and remotely across the many time zones. We were called on to support the needs of our constituents by providing them with extra support and advising during the pandemic. This was accomplished because of the “time tested” MW CTR-IN integrated organizational structure and process.

Recommendations in the future include (1) continued outreach to 13 institutions using remote online access in ATRIP, NMRN, and GWW, (2) continued use of “personal diplomacy,” (3) use of the MW CTR-IN portal to reach MW CTR-IN constituents in 13 institutions via news and updates of MW CTR-IN, (4) opening a wider catchment to MW CTR-IN nonfaculty using a special portal for usage of MW education research resources, and (5) continued improvement to upgrade tracking and evaluation data using new portal software.

## Summary

In summary, MW CTR-IN PD core activities are current with innovative teaching and learning approaches and reflect evidence-based practice in education and career development. Our regional PD core continues to serve our 13 institutions among seven IDeA states targeting health disparities. Using these best practice evidence-based strategies have been successfully implemented in our MW CTR-IN research infrastructure regional network to address barriers and metrics. Applying an educational conceptual model by Fink (2013) has grounded best educational practice approaches and provides a sound foundation with implications for future use in other CTR-IN networks. The PD core model may have limitations based on the size of the program, but we believe the MW CTR-IN program has invested in a wise decision to decentralizing rather than centralizing our resources and services to reach individual institutions, and we hope to prove this by the end of the second 5-year period of this grant.
